# Investigating the impact of fluid status on the ultrasound assessment of muscle quantity and quality in the diagnosis of sarcopenia – a multidimensional cross-sectional study

**DOI:** 10.1186/s12877-023-04177-6

**Published:** 2023-08-15

**Authors:** Benjamin Stanley, Carolyn Greig, Thomas Jackson, Danielle Lewis, Hannah Moorey, Zainab Majid, Tahir Masud, Thomas Pinkney, Carly Welch

**Affiliations:** 1 Northern Care Alliance NHS Foundation Trust, Salford Royal, Stott Lane, Salford, M6 8HD UK; 2 Medical Research Council – Versus Arthritis Centre for Musculoskeletal Ageing Research, University of Birmingham and University of Nottingham, Nottingham, UK; 3 School of Sport, Exercise, and Rehabilitation Sciences, University of Birmingham, Birmingham, B15 2TT UK; 4 National Institute for Health Research Birmingham Biomedical Research Centre, University of Birmingham and University Hospitals Birmingham NHS Trust, Birmingham, UK; 5 Institute of Inflammation and Ageing, College of Medical and Dental Sciences, University of Birmingham, Birmingham, B15 2TT UK; 6 Department of Healthcare for Older People, University Hospitals Birmingham NHS Foundation Trust, Birmingham, B15 2GW UK; 7 Queen’s Medical Centre, Nottingham University Hospitals NHS Trust, Nottingham, NG7 2UH UK; 8 School of Medicine, University of Nottingham, Nottingham, NG7 2UH UK; 9 Academic Department of Surgery, University Hospitals Birmingham NHS Foundation Trust, Birmingham, B15 2GW UK; 10 Institute of Applied Health Research, College of Medical and Dental Sciences, University of Birmingham, Birmingham, B15 2TT UK; 11 Guy’s and St Thomas’ NHS Foundation Trust, London, UK

**Keywords:** Sarcopenia, Ultrasound muscle assessment, Echogenicity, Bioelectrical Impedance Analysis

## Abstract

**Background:**

Sarcopenia is a clinical manifestation of adverse ageing, characterised by progressive loss of muscle mass and function. Diagnosis requires assessment of muscle quantity and quality; ultrasound represents an emerging tool for this. However, ultrasound muscle assessment may be impacted by fluid balance. This is particularly important when assessing for acute sarcopenia in hospitalised patients, where fluid disturbance often occurs. The primary aim of this study was to characterise the impact of fluid status on ultrasound muscle assessment, such that this may be accounted for in sarcopenia diagnostics.

**Methods:**

This Multidimensional Cross-sectional study involved 80 participants, who were inpatients at QEHB, a large UK tertiary centre. Fluid status was evaluated clinically and quantified using Bioelectrical Impedance Analysis (BIA). Muscle quantity was measured using Bilateral Anterior Thigh Thickness (BATT) with Rectus Femoris (RF) echogenicity used to assesses muscle adiposity and hence provide an inverse measure of muscle quality.

**Results:**

A significant positive correlation was found between fluid status, measured using BIA, and BATT as a measure of muscle quantity, in males (rs = 0.662, p < 0.001) and females (rs = 0.638, p < 0.001). A significant negative correlation was found between fluid status and RF echogenicity (rs=-0.448, p < 0.001).

**Conclusions:**

These findings demonstrate associations between fluid balance and ultrasound assessment of muscle quantity and quality. Given the emerging use of ultrasound muscle assessment in sarcopenia diagnosis, there is a need to account for this in clinical practice. Future research should focus on the development of a corrective equation allowing assessment of muscle quantity and quality which account for changes in fluid status, hence aiding accurate diagnosis of sarcopenia.

## Background

Sarcopenia is a condition of muscle insufficiency defined by reduced muscle strength in combination with reduced muscle quantity or quality [1]. Sarcopenia is closely related to ageing with the prevalence increasing with age both in the community [2] and amongst hospitalised patients [3]. It is associated with increased risk of falls [4], reduced quality of life [4], and all-cause mortality [5]. Additionally, sarcopenia has been demonstrated to be associated with a reduction in mobility in terms of gait speed and Timed-up-and-go-test performance [6]. It should also be noted that sarcopenia is often associated with other conditions related to ageing, such as nutritional deficiency [7], and osteopenia/osteoporosis [8].

Acute sarcopenia is an emerging concept which refers to the loss of muscle mass and function following a ‘significant physiological stressor’ [9] such as acute illness or surgery [9]. The EWGSOP2 group introduced acute sarcopenia into the revised definition of sarcopenia in 2019, defining it as the development of sarcopenia in fewer than 6 months, commonly associated with acute illness or injury [1].

Computed Tomography (CT), Magnetic Resonance Imaging (MRI), and Dual-energy X-ray Absorptiometry are recommended as gold-standard techniques for muscle quantity, [1] but each have limitations when performed serially, and are not feasible to perform at the bedside or outside of the hospital. Ultrasound assessment of muscle offers a potential solution in that is portable, allowing bedside assessment [10]; inexpensive [11]; easily accessed clinically [12]; and does not expose patients to ionising radiation [11]. A major advantage of the use of ultrasound in muscle assessment is the ability to assess muscle quality in addition to muscle quantity [1].

Ultrasound assessment of muscle quality is performed by assessment of echogenicity; a tissue’s ability to reflect or absorb ultrasound energy [13]. Adipose tissue is hyperechoic (white), meaning greyscale analysis can be used to produce values for echogenicity which represent intramuscular adiposity, and hence inversely reflect muscle quality [10]. There is, however, emerging evidence that ultrasound measures of muscle quantity [14, 15] and quality [16, 17] are affected by changes in fluid status.

Hospitalised patients, in addition to being at risk of acute sarcopenia, are also at increased risk of disturbance to fluid physiology [18]. Fluid status in hospitalised patients is most commonly assessed clinically [19] using classically described signs and symptoms of hypo-/hypervolaemia [20], despite the poor evidence base underlying these [21].

Bioelectrical Impedance Analysis (BIA) is an additional method which may be used to assess patients’ fluid status. BIA is based on the property of impedance; defined as the obstruction of flow of an alternating current [22]. Using electrodes, most commonly placed on the right hand and right foot [22], a small alternating current is applied, and resistance is measured, allowing impedance to be calculated [23]. Comparison of impedance measurements to reference values, accounting for age, sex and BMI, then allows Total Body Water to be calculated [24].

## Methods

### Objectives

1. To assess the relationship between clinical balance assessment and total body water quantified by bioelectrical impedance analysis.
2. To assess the relationship between total body water quantified by bioelectrical impedance analysis with muscle quantity and quality assessed using ultrasonography.

### Patient recruitment

This Multidimensional Cross-sectional study involved 80 participants, who were inpatients at QEHB, a large UK tertiary centre.

This work constitutes a sub-study of a wider project characterising acute sarcopenia in older adults [25]. Participants were recruited from the Queen Elizabeth Hospital Birmingham (QEHB). Three distinct groups were recruited: Patients undergoing elective colorectal surgery (‘Elective’), patients undergoing emergency abdominal surgery (‘Emergency surgery’), and medical inpatients admitted with confirmed or suspected acute bacterial infection (‘Medical’).

Eligible patients were those 70 years or older at the time of recruitment with the above admissions. Exclusion criteria for the study were elective patients unable to provide written consent, patients unable to understand verbal English, patients who were immobile prior to hospital admission, and those with a life expectancy < 30 days.

Eligible patients were initially approached either in pre-operative assessment clinic (elective patients) or post-admission (emergency surgery and medical patients) and given verbal and written information on the study. They were then given time to consider participation and re-visited. If patients wished to participate, they were recruited at this stage.

**Fig. 1:**
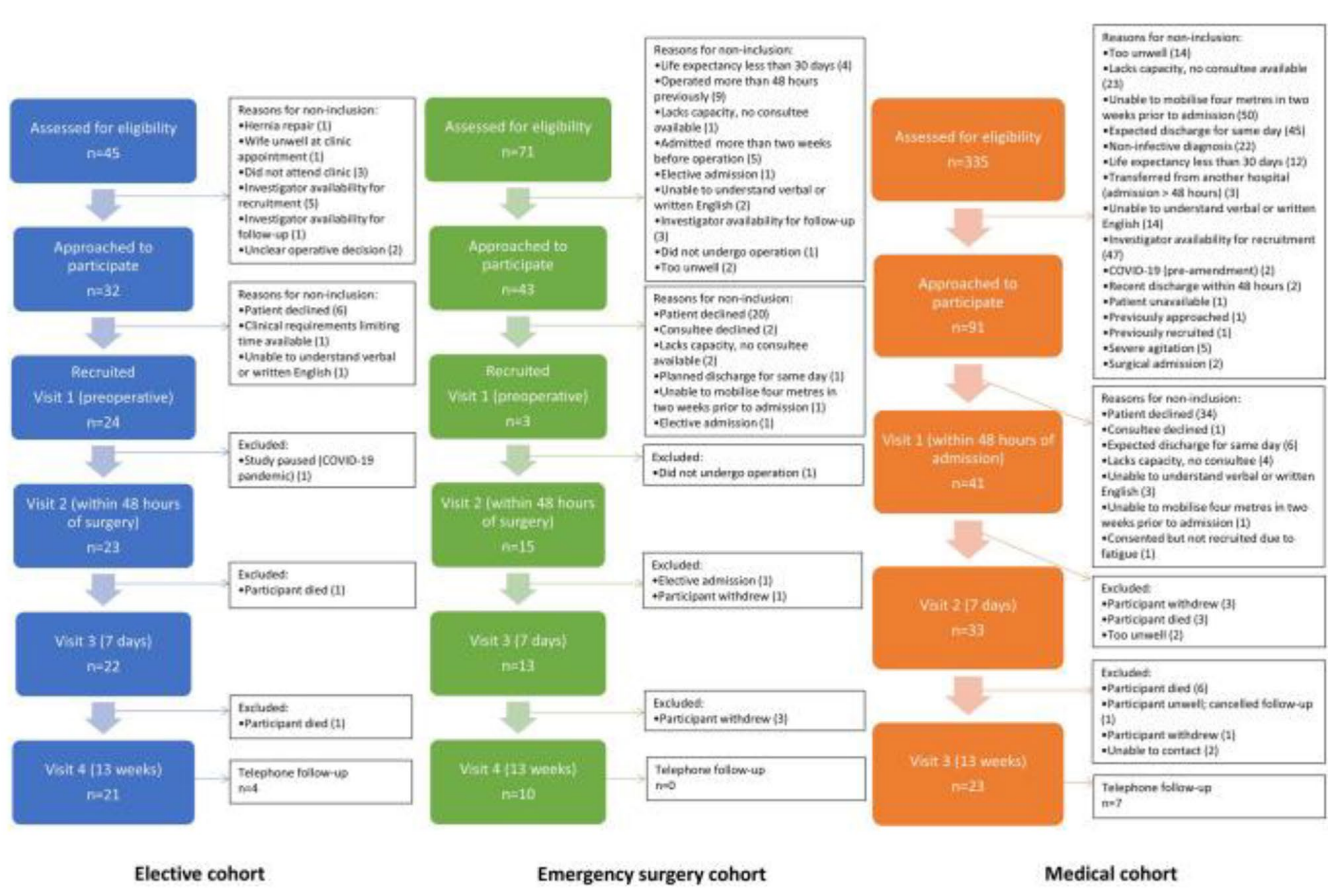
Screening, recruitment, and follow-up rates for participants in all cohorts and reasons for non-participation

Figure 1, as initially published in the feasibility protocol [25] and replicated below, illustrates numbers of patients screened, approached, recruited and those lost to follow-up.

All elective patients provided written informed consent to study participation. These patients were asked if they would wish to remain in the study should they lose capacity post-operatively, for example due to delirium. All patients agreed that they would. Medical and emergency surgery patients also provided written informed consent where able. If these patients lacked capacity, legally authorised representative was able to declare the patients’ wish to participate, with the aim of avoiding patient exclusion due to transient loss of capacity.

Initial assessment for elective patients was conducted within the pre-operative assessment clinic (Visit 1). Further assessments then took place 48 h (Visit 2), 7 days (Visit 3) and 13 weeks (Visit 4) post-surgery. The aim was to recruit and assess emergency patients preoperatively (Visit 1), although recruitment and initial assessment were conducted within 48 h post-surgery (Visit 2) when preoperative assessment was not possible. Further assessments were conducted 7 days (Visit 3) and 13 weeks (Visit 4) post-surgery. Medical patients were recruited and initially assessed in the first 48 h after admission (Visit 1), with further assessments at 7 days (Visit 2) and 13 weeks (Visit 3) post-admission.

### Ethical approval

This study has obtained ethical approval from Wales REC 4 (19/WA/0036) and the Health Research Authority.

### Assessment of fluid status

Fluid status was assessed at each assessment both clinically and using Bioelectrical Impedance Analysis (BIA).

Clinical assessment of fluid status was performed by an experienced geriatric doctor working as a senior registrar at the time of data collection (CW). These assessments were not blinded by nature. Fluid status was defined as clinically hypovolaemic, clinically euvolaemic, or clinically hypervolaemic. This was based on holistic assessment of clinical signs and symptoms, and assessment of fluid input and output based on chart recordings. Signs and symptoms assessed included skin turgor, presence of pedal/sacral oedema and/or ascities, mucous membranes, capillary refill time and Jugular Venous Pressure (JVP).

BIA measurements were carried out using a Bodystat Quadscan 4000 analyser (Bodystat Ltd, Isle of Man, British Isles), operated by CWThis allows Multi-Frequency BIA (MF-BIA) and hence determination of Extracellular Water (ECW), Intracellular Water (ICW), and Total Body Water (TBW) [26]. To perform BIA, four electrodes were used, with two placed on the right hand and two on the right foot. The electrodes on the hand were placed on the dorsum of the hand, proximal to the metacarpal-phalangeal joints, and on the ulnar aspect of the posterior wrist. The electrodes on the foot were placed on the dorsum proximal to the metatarsal-phalangeal joints, and on the anterior aspect of the ankle between the malleoli. Raw values of impedance, resistance and reactance were produced, in addition to calculated values of TBW, ECW, ICW, third space, fluid volume, fat weight and lean weight.

### BIA assessment of skeletal muscle mass

A previously validated equation; SMM = − 3.964 + (0.227 x (height2/ resistance)) + (0.095 x weight) + (1.384 x sex) + (0.064 x reactance) [27] was used to calculate Skeletal Muscle Mass (SMM) in the study population. In order to account for differences in height between participants, index values for SMM will be used, calculated as SMM/height2.

### Ultrasound assessment of muscle

Ultrasound assessment of muscle quantity and quality was performed at each patient visit. Muscle quantity was assessed using Bilateral Anterior Thigh Thickness (BATT), with muscle quality assessed by rectus femoris echogenicity. Participants sat at 45°, with knees flexed at 10–20°. The ultrasound probe was placed at the midpoint of the thigh, as identified by measuring the distance between the greater trochanter and the lateral joint line of the knee. Images for both the right and left thigh were recorded. Scanning itself was performed by CW and image analysis was conducted by both CW and BS, at the time an intercalating medical student, who were blinded at the point of analysis.

### Calculation of bilateral anterior thigh thickness

**Fig. 2:**
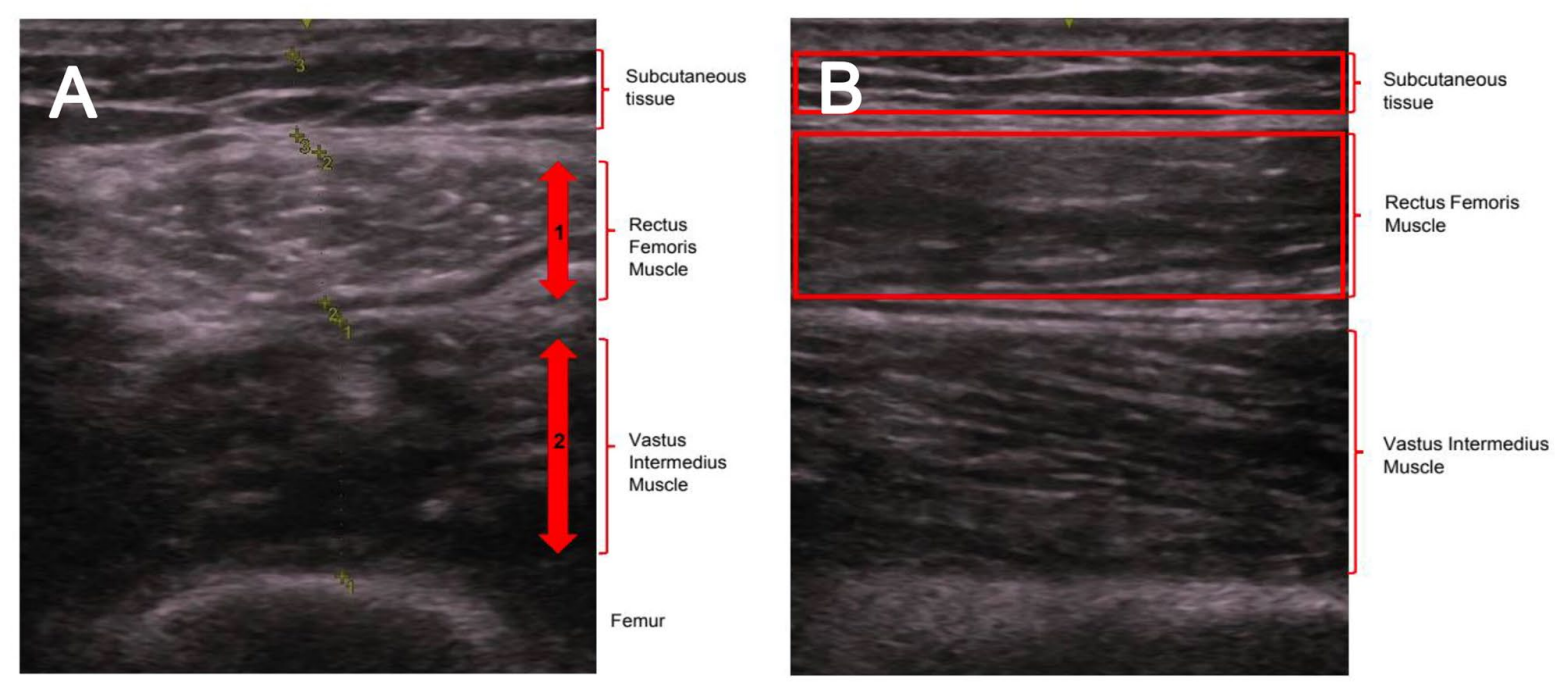
**A**) measurement of BATT in transverse plane. **B**) measurement of echogenicity in longitudinal plane. (**A**) The thickness of the Rectus Femoris (1) and of the Vastus Intermedius (2) were measured and summed. Totalling of this value bilaterally gives an overall value for BATT, recorded in cm. (**B**) Subcutaneous (SC) tissue, Rectus Femoris (RF) muscle and Vastus Intermedius (VI) muscle are identified. Boxes were then drawn surrounding the SC and RF as shown, with greyscale analysis function within ImageJ used to calculate echogenicity

Images used in the calculation of BATT were taken in the transverse plane. The thickness of the Subcutaneous (SC) tissue, Rectus Femoris (RF) muscle and Vastus Intermedius (VI) muscle was measured, not including fascial planes. This is demonstrated in Fig. 2. BATT was then calculated as the total thickness of the two muscles (RF and VI) bilaterally [28]:

$$\begin{array}{ccccc}BATT \left( {cm} \right) & = Right\,RF\,thickness\\&+ Left\,RF\,thickness\\&+ Right\,VI\,thickness\\&+ Left\,VI\,thickness\end{array}$$

As muscle quantity has sex-specific cut-offs within the diagnostic guidelines [1], analyses for male and female participants involving these measures were performed separately.

### Assessment of echogenicity

Assessment of echogenicity was performed using Longitudinal images. The echogenicity of SC and RF were assessed by greyscale analysis using ImageJ software [29], as demonstrated in Fig. 2. Measures for both right and left sides were performed, with mean values used for analysis.

### Statistical analysis

Data management and transformation was conducted in Microsoft Excel 2019 (Microsoft Corporation, Redmond, WA, USA) with statistical testing conducted in RStudio Version 1.3.1093 (2020) (PBC, Boston, MA, USA). Figures were generated using Graphpad Prism Version 9.0.2 (GraphPad Software, La Jolla, Ca, USA).

Normality of data distributions was assessed statistically using the Shapiro-Wilk test and visually using Q-Q plots. Where continuous variables were found to be normally distributed, summary statistics presented are mean values with standard deviation. Where continuous variables did not follow a normal distribution, summary statistics are presented as median values with interquartile ranges to avoid distortion of mean values by outliers. Differences between normally distributed continuous variable were assessed for significance using one-way analysis of variance (ANOVA) with Tukey post-hoc testing.

Where continuous variables were not normally distributed, differences were assessed for significance using the non-parametric Kruskal-Wallis test with Dunn’s post-hoc testing. The Bonferroni correction was applied to account for multiple testing. Differences between ordinal categorical variables were assessed for significance using the Kruskal-Wallis test, with the chi-squared test used to assess the significance of differences between nominal categorical variables.

Correlation between continuous variables was assessed using the Pearson correlation coefficient where both variables were normally distributed, with the non-parametric Spearman rank correlation coefficient calculated where one or more variable was not normally distributed. Multivariable analyses were not performed.

The level of statistical significance for all tests was set at 9 5%.

## Results

### Patient recruitment

A total of 80 patients were recruited to the study; 24 in the elective group, 15 in the emergency surgery group, and 41 in the medical group. Loss to follow-up was 12.5% in the elective group, 33.3% in the emergency surgery group, and 41.5% in the medical group. Previously published feasibility results [25] outline numbers recruited and the number of assessments performed at each stage. The assessments completed at each visit have been pooled when assessing the impact of fluid status on the ultrasound assessment of muscle quantity and quality.

### Demographics

**Fig. 3:**
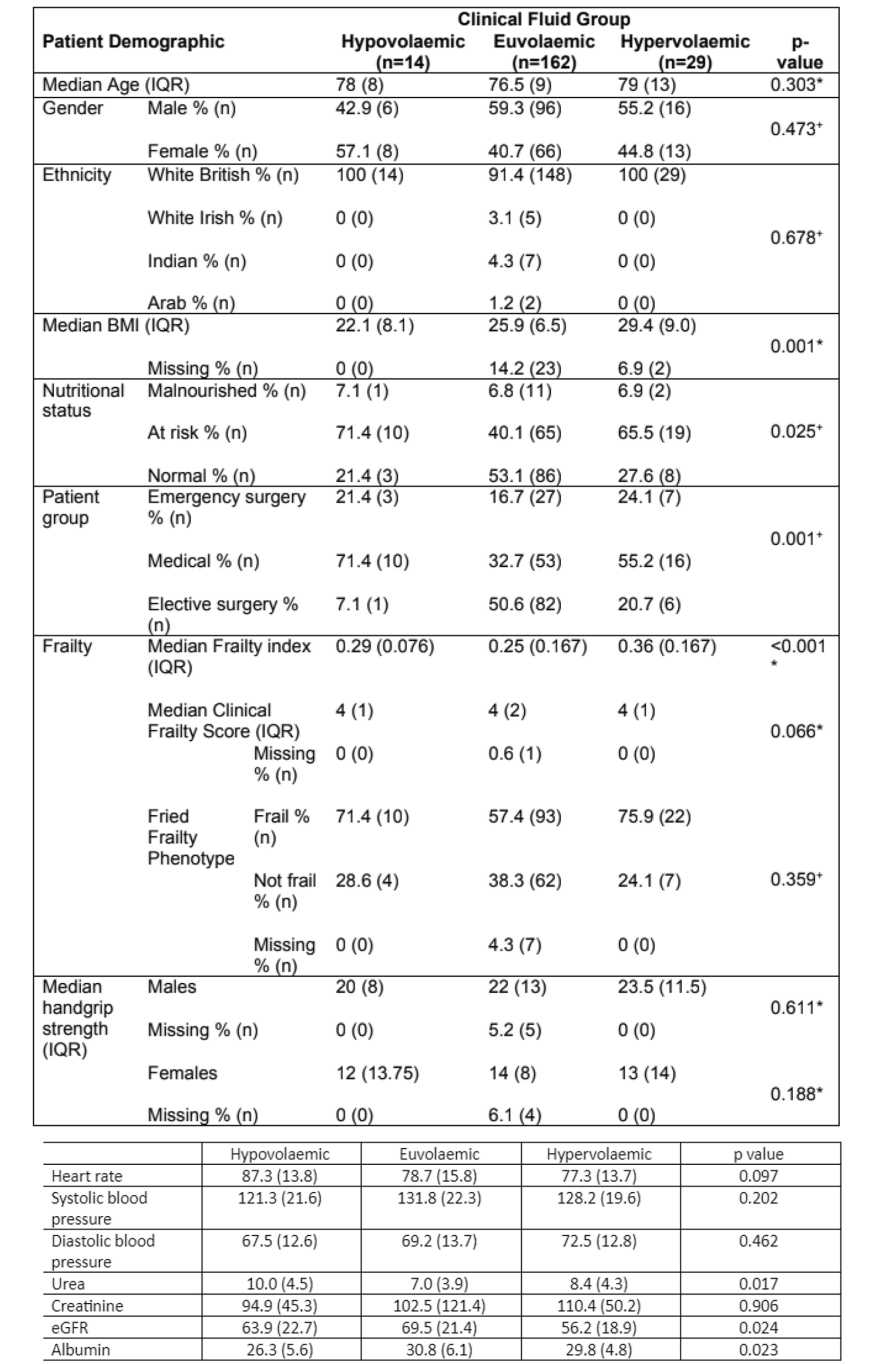
Summary of data acquired at patient assessments across all visits, divided by clinical fluid status. Hypovolaemic n = 14, Euvolaemic n = 162, Hypervolaemic n = 29. Nominal variables (gender, ethnicity, patient group, nutritional status and Fried Frailty Phenotype) are presented as percentage values and raw n values. Clinical Frailty Score, an ordinal variable, is summarised using median and interquartile range. Continuous data was tested statistically for normality using the Shapiro-Wilk test. Data for age, BMI, Frailty Index and handgrip strength did not follow a normal distribution and are therefore summarised as median values with interquartile ranges. * = p-value generated using Kruskal-Wallis test, + = p-value generated using Pearson’s chi-squared test

Figure 3 shows the demographic data of participants, according to clinical fluid status. A total of 205 patient assessments where clinical fluid status was recorded were performed. At 6.8% (n = 14) of these assessments the patient was clinically hypovolaemic, 79.0% (n = 162) involved clinically euvolaemic patients, and 14.1% (n = 29) involved clinically hypervolaemic patients.

### Assessment of bioelectrical impedance analysis as a quantification of fluid status

Differences in BIA measurements of fluid status were compared between clinical fluid groups to assess the validity of BIA as a quantification of clinical fluid status in the study population. A total of 178 patient assessments were performed where both clinical fluid assessment and BIA measurements were conducted; 7.3% (n = 13) of these patients were clinically hypovolaemic, 78.7% (n = 140) were clinically euvolaemic, and 14.0% (n = 25) were clinically hypervolaemic.

**Fig. 4:**
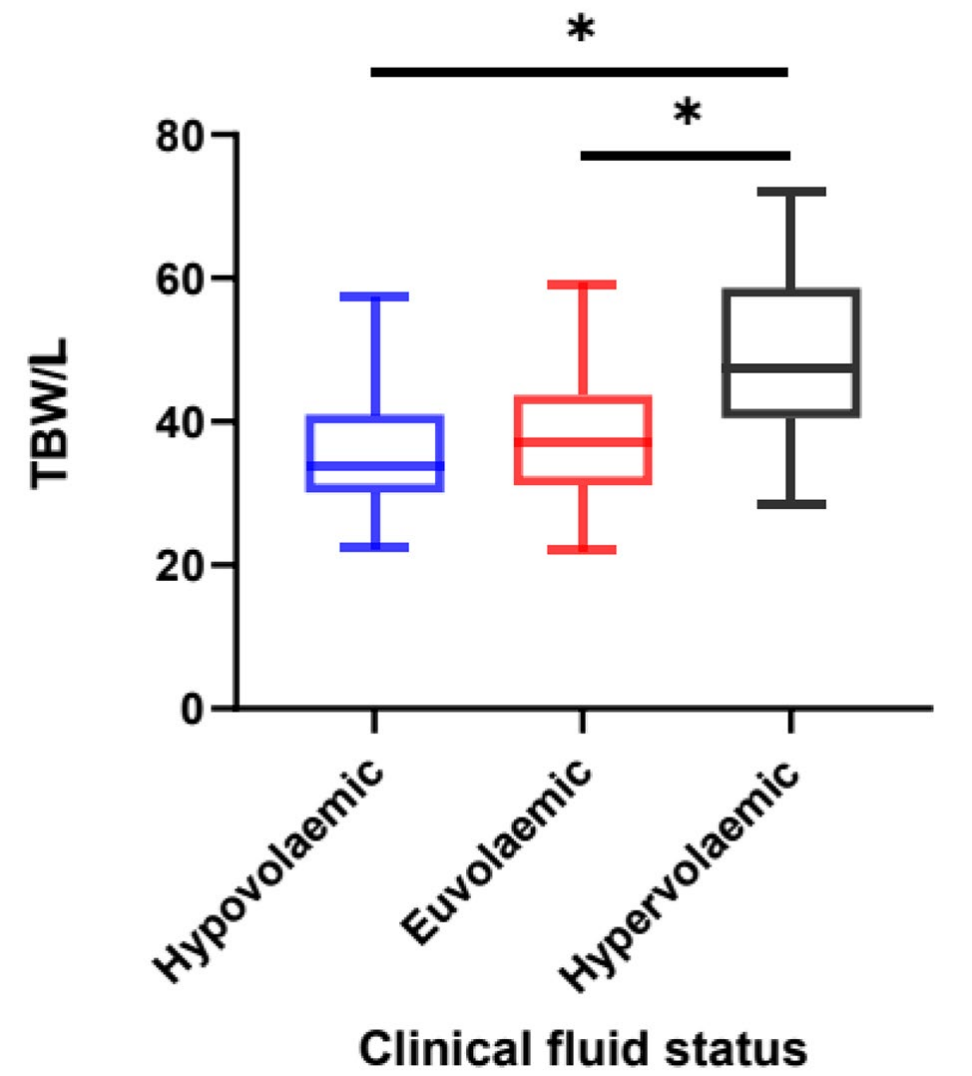
Box and Whisker plot demonstrating Total Body Water divided by clinical fluid status. Data acquired from BIA measurement and clinical assessment of fluid status across all patient assessments from all visits. Hypovolaemic n = 13, Euvolaemic n = 140, Hypervolaemic n = 25. The centre of each box represents the median value, with the box representing the IQR and the ‘whiskers’ representing minimum and maximum values. A non-parametric Kruskal-Wallis test was used to compare the differences between groups as the data was not normally distributed, with the Dunn’s test applied post-hoc. * = p < 0.05

Median TBW was lowest in the clinically hypovolaemic group (33.9 L) compared to the clinically euvolaemic (37.1 L) and clinically hypervolaemic (47.3 L) groups. A Kruskal-Wallis test found a statistically significant difference (χ2 (df = 2) = 17.46, p < 0.001) between these groups. A post-hoc Dunn’s test demonstrated a statistically significant difference between the clinically hypervolaemic group and both the clinically euvolaemic (p < 0.001) and the clinically hypovolaemic (p = 0.008) groups. No significant difference was found between the clinically hypovolaemic and euvolaemic groups. This data is demonstrated in Fig. 4.

### Assessment of validity of BATT as a measure of skeletal muscle mass

**Fig. 5:**
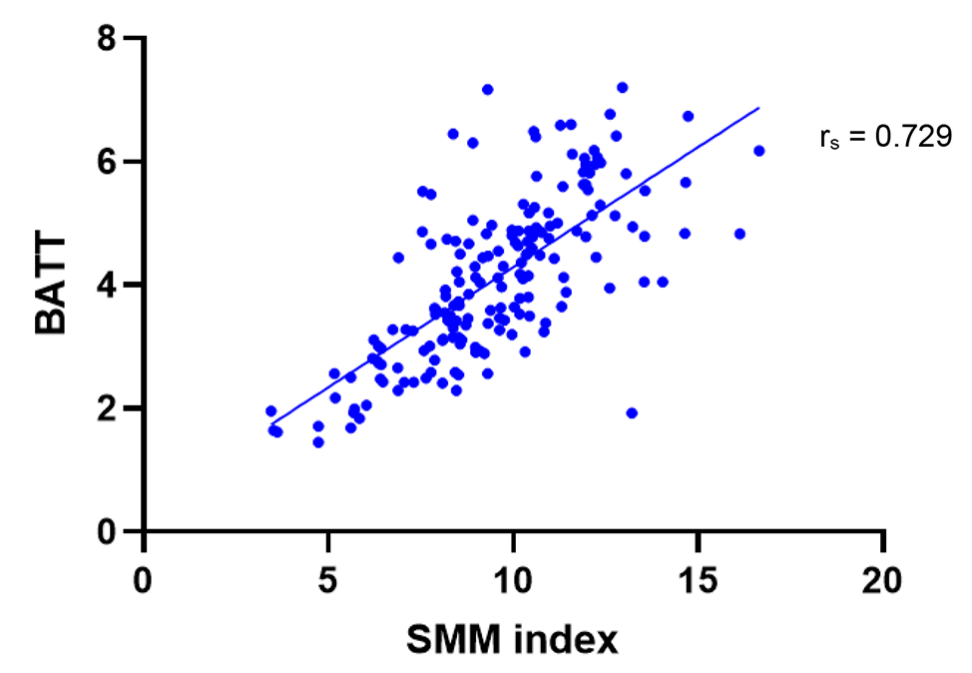
Bilateral Anterior Thigh Thickness (BATT) in comparison to Skeletal Muscle Mass (SMM) index. BATT values acquired from ultrasound assessment of quadriceps muscles. SMM index values obtained from BIA assessment. All patient assessments included. n = 180. Correlation coefficient calculated using non-parametric Spearman rank correlation

In order to assess the accuracy of using BATT as a measure of skeletal muscle mass, BATT values were compared with index SMM values estimated via BIA. Data was available for 180 patient assessments where both variables were calculated. A strong positive correlation (r_s_ = 0.729, 95% CI: 0.650–0.792, p < 0.001) was found between these variables, as indicated in Fig. 5.

### Bilateral anterior thigh thickness in comparison to bioelectrical impedance analysis of fluid status

**Fig. 6:**
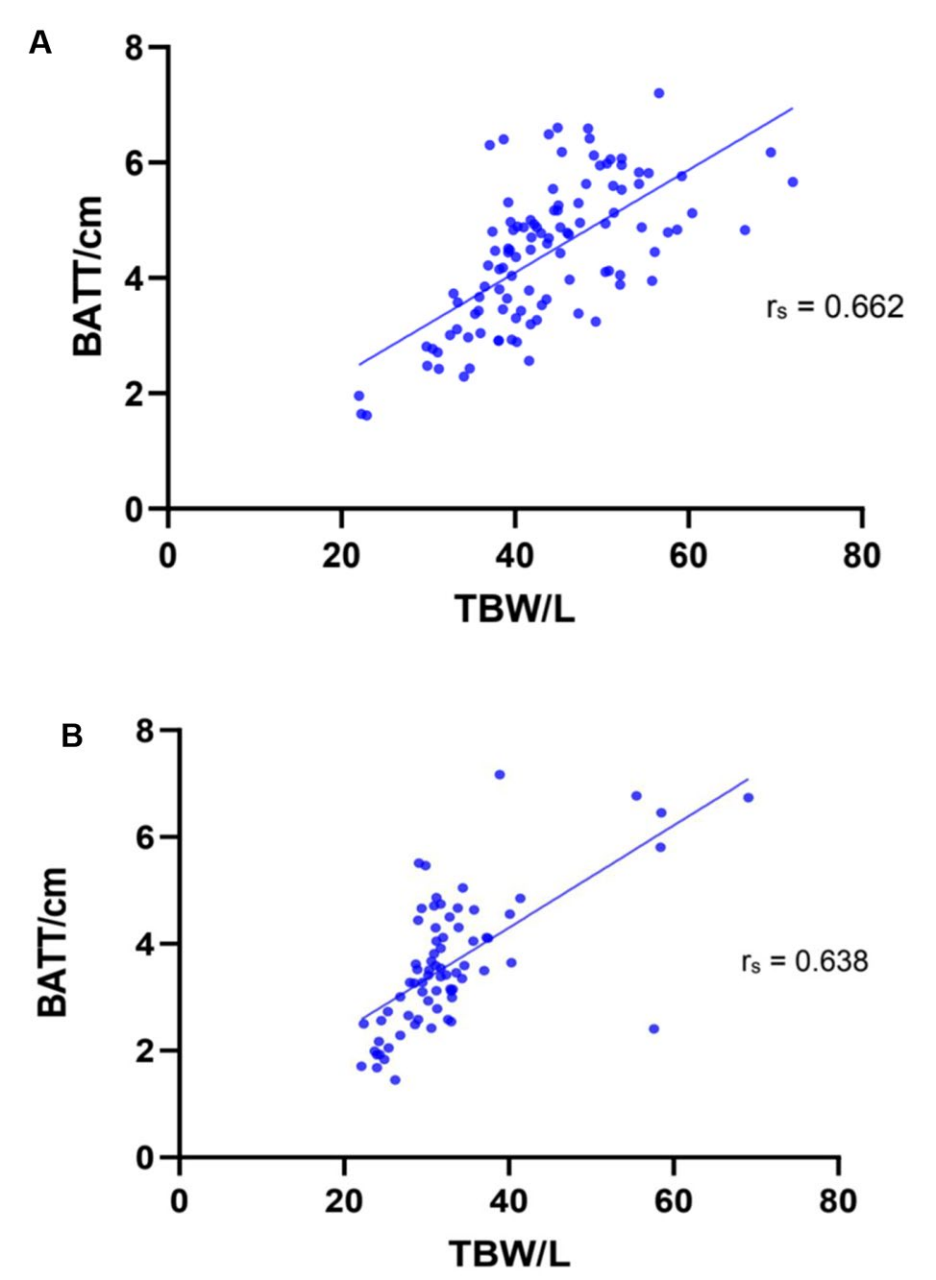
Total Body Water and Bilateral Anterior Thigh Thickness in Males (**A**) and Females (**B**). TBW values acquired from BIA assessment. BATT values acquired from ultrasound assessment of quadriceps muscles. Patients across all patient groups and across all visits are included. (**A**) n = 107. (**B**) n = 73. Correlation coefficient calculated using non-parametric Spearman rank correlation

Data were available for 107 assessments in males, and 73 in females, where both BATT was recorded using ultrasound assessment, and TBW was recorded using BIA. As muscle quantity has sex-specific cut-offs within the diagnostic guidelines [1], analyses for male and female participants were performed separately. A strong positive correlation was found between TBW and BATT in both males (rs = 0.662, 95% CI: 0.536–0.759, p < 0.001) and females (rs = 0.638, 95% CI: 0.472–0.760, p < 0.001). Figure 6 visualises this data.

### Rectus femoris echogenicity in comparison to clinical fluid status

**Fig. 7:**
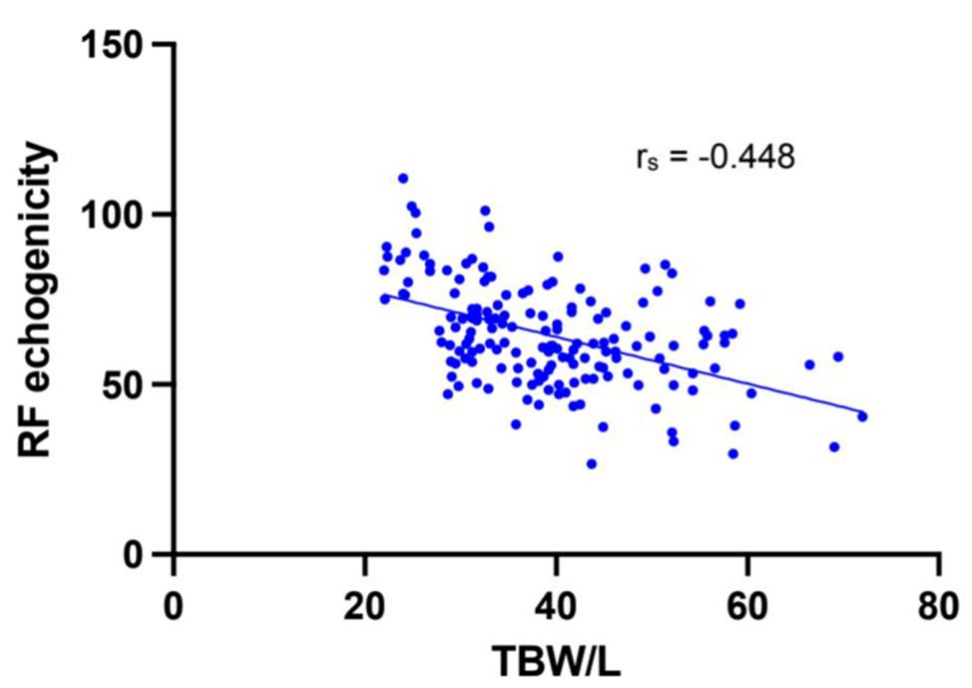
Scatter plot of Total Body Water and Rectus Femoris echogenicity. TBW values acquired from BIA assessment. RF echogenicity calculated from ImageJ greyscale analysis of longitudinal ultrasound images. All patient assessments included. n = 162. Correlation coefficient calculated using non-parametric Spearman rank correlation

A total of 162 patient assessments were performed which included both BIA, to assess TBW, and ultrasound, to assess echogenicity. A moderate negative correlation was found between RF echogenicity and TBW (rs = -0.448, 95% CI: -0.567 – -0.312, p < 0.001; Fig. 7).

## Discussion

Sarcopenia represents a significant burden both to individuals and to healthcare systems [30]. The diagnosis of sarcopenia is challenging, with the assessment of muscle quantity and quality in particular presenting difficulties for the field [1]. Ultrasound represents an emerging non-invasive method of assessing muscle [10]. However, pilot studies and findings from the literature suggest these measures may be impacted by changes in fluid status [14], which are particularly prevalent in hospitalised older adults at risk of acute sarcopenia. The primary aim of this research was therefore to evaluate the effect of changes in fluid status on the ultrasound assessment of muscle quantity, assessed using the validated BATT measure, and muscle quality, assessed using Rectus Femoris echogenicity.

A strong positive correlation was found between TBW, as assessed by BIA, and BATT, in both male and female patients. This supports the hypothesis that increased fluid volume correlates with an increase in the measurement of muscle quantity by ultrasound and provides potential reasoning for the postoperative increases in BATT seen in the pilot study which prompted this work [14].

This finding was novel in that it is the first comparison of ultrasound assessment of muscle quantity with quantified in vivo fluid status. It is also consistent with previous literature; Fischer et al. [15] found a positive correlation between fluid balance (as assessed by input and output monitoring) and Muscle Layer Thickness (MLT), an alternative measure to BATT which also measures muscle quantity via ultrasound assessment of the quadriceps.

The second domain of ultrasound muscle assessment analysed in regard to the impact of fluid status was muscle quality, as measured by echogenicity. Echogenicity reflects the adiposity of muscle, and is hence inversely related to muscle quality. A moderate negative correlation was found between TBW and RF echogenicity, supporting the hypothesis that an increase in fluid volume correlates with a decrease in muscle echogenicity.

This finding is consistent with expected changes in ultrasound imaging. Fluid is hypoechoic and therefore appears grey/black on ultrasound [13]. Hence, when the volume of fluid within muscle increases, quantification of echogenicity with greyscale analysis produces lower relative values, leading to the negative correlation seen.

This study represents the first assessment of muscle echogenicity in comparison to in vivo fluid quantification, hence literature on the subject is limited. Previous work has found unexplained variation in RF echogenicity within a cohort of critically unwell patients [17] and the present finding of a correlation between fluid status and echogenicity may offer an explanation for this. However, Puthucheary et al. [16] found no correlation between fluid balance and RF echogenicity in a cohort of 30 critically unwell patients. As recognised by the authors however, monitoring of fluid balance does not accurately reflect in vivo fluid status [16]. Hence, this present study, where fluid status was more accurately assessed using BIA, more likely represents a true measure of fluid volume and hence the correlation found with echogenicity can be concluded to be a true one.

### Strengths and limitations

A significant challenge in incorporating ultrasound muscle assessment into diagnostic protocols for sarcopenia has been the difficulty in setting standardised cut-offs given the heterogeneity in specific muscle assessment and patient positioning [11]. Hence, a major strength of this study was the use of a standardised protocol [28] in the assessment of both muscle quantity using BATT and muscle quality using echogenicity, as this accurately contextualises the impact of changing fluid status on ultrasound muscle assessment.

An additional strength to this study was the representative nature of the patient population included. Medical, elective surgical, and emergency surgical patients were all included with the aim of accurately reflecting the population of hospitalised older adults. This is in comparison to other literature in the field which often focuses solely on acutely unwell or surgical patients only.

Furthermore, patients were not excluded from the study due to a transient loss of capacity. All elective patients gave their consent for continued study inclusion should their capacity become impaired, legally authorised representative was able to declare the wish to participate of a medical or emergency surgery patient who lacked capacity. Given the prevalence of delirium in hospitalised patients has been estimated to be as high as 50% [31], patients with a transient loss of capacity represent a significant proportion of hospitalised older adults. These patients are often excluded from clinical research due to the potential challenges involving consent [32], so the fact they were included here represents a strength in terms of ensuring a representative study population.

It must be noted however that the study population was not representative of the wider population in terms of ethnicity, with the vast majority of patients being white British. The under-representation of ethnic minorities in clinical research is a recognised problem [33] which is not isolated to this research. It is of particular concern here however, as unpublished data from the research group demonstrates that ultrasound assessments of muscle quantity and quality are affected by ethnicity, so a representative study population is particularly key in ensuring accurate conclusions are drawn on the impact of fluid status on these measures. An exclusion criterion for this study was inability to understand written English and it is possible this contributed to the under-representation of patients from ethnic minorities.

All clinical, BIA and ultrasound assessments were carried out by the same clinician to ensure consistency of measurement given the significant inter-operator variability of these assessments, particularly in older adults [34, 35]. Conversely, greyscale analysis of echogenicity using ImageJ was performed by two different assessors, given the volume of images requiring analysis. This assessment also shows significant inter-user variability [23], hence this is a recognised limitation.

A further limitation of this study was the quantification of fluid status with BIA, in patients where fluid balance was disturbed. The accuracy of BIA in this setting is disputed [36]. Hence, although it was a suitable measurement tool here given ease of use and patient acceptability [24], it does not represent the gold standard method of fluid balance quantification. This would entail isotope dilution assessment [37], however the costs, time intensity and lack of tolerability render this inappropriate in the study setting [37].

### The impact of these findings on clinical practice

This study represents the first work demonstrating correlation between quantified in vivo fluid status and changes to muscle quantity and quality as assessed by ultrasound. This is an important finding with clear relevance to the field of sarcopenia diagnostics.

Ultrasound assessment represents an accurate, accessible, and safe method of assessing muscle quantity [1]. Therefore, ultrasound muscle assessment is likely to become a fixture in sarcopenia diagnosis. Hence, the finding that BATT measures are impacted by the fluid status of a patient is important and demonstrates the need to account for changes in fluid balance to ensure the accurate assessment of muscle quantity, given its importance in the diagnosis of sarcopenia.

There is also increasing recognition that muscle function is related not only to muscle quantity, but also to the quality of the muscle [38–40]. As such, accurate assessment of muscle quality has been identified by EWGSOP2 [1] as a key area of future study within the field of sarcopenia. This present study demonstrates that in addition to muscle quantity, ultrasound assessment of muscle quality, in the form of echogenicity, is also impacted by changes in fluid status, demonstrating the need to account for this in diagnostic assessment.

These findings are of particular importance in the context of acute sarcopenia in hospitalised older adults, as this is the context in which acute changes to fluid status are most likely [18]. Hence, ultrasound muscle assessments in this setting are more likely to be impacted by changes in fluid status compared to assessment of chronic sarcopenia in the community. Therefore, it is in this subset of sarcopenia diagnostics that these findings are likely to be most impactful.

Ensuring the accurate diagnosis of sarcopenia is vital, particularly in the emerging group of patients at risk of acute sarcopenia secondary to hospitalisation. Accurate diagnosis allows further research, such as that on potential interventions, to be more effectively targeted [41]. Furthermore, early evidence demonstrates the potential benefits of neuromuscular electrical stimulation (NMES) and early mobilisation in these patients [42], so accurate early diagnosis has the potential to have a direct positive influence on patient outcomes. Given the impact changes in fluid status may have on the accuracy of diagnosis as discussed, these benefits of early diagnosis further reinforce the importance of accounting for this when assessing muscle quantity and quality using ultrasound.

### Future research

The findings of this study should be considered by future research in the field of sarcopenia diagnostics. This should consider the impact of fluid status on ultrasound measures of muscle quantity and quality, particularly in the context of acute sarcopenia secondary to hospitalisation.

This study demonstrated correlations between fluid status and measures of muscle quantity and quality using ultrasound assessment. Although it is likely that the direction of causality here is changing fluid levels leading to a change in muscle assessment parameters, the correlation presented does not prove this. Therefore, the most immediate next step in research should be confirmation of a causative relationship between changes in fluid status and changes in BATT and echogenicity.

In order for these findings to have a tangible impact on clinical practice, there is a need for the development of a corrective equation that provides values for BATT and echogenicity which take into account any disturbance in fluid balance, assessed most likely by BIA, given this has been found to accurately quantify fluid status in hospitalised older adults. This would then generate values for BATT and echogenicity which more accurately characterise true muscle quantity and quality, given the impact of fluid status would be negated, hence improving the accuracy of sarcopenia diagnoses, and therefore improving patient outcomes.

## Conclusion

Whilst two of the three criteria of sarcopenia diagnosis, muscle strength and physical.

performance, can be easily assessed in a clinical setting, assessment of muscle quantity and quality remains a barrier to accurate diagnosis. Ultrasound assessment of muscle provides a potential solution to this problem, allowing accurate, safe and accessible assessment of both muscle quantity and quality. The results of this study suggest that disturbance in fluid status impacts this ultrasound assessment, specifically in the context of acute sarcopenia in hospitalised patients over the age of 70. Future research should focus on the development of a corrective equation which accounts for fluid status in determining values of muscle quantity and quality. This would allow more accurate diagnoses of sarcopenia to be made, therefore facilitating potential early intervention and hence improving patient outcomes.

## Data Availability

The datasets used and/or analysed during the current study are available from the corresponding author on reasonable request.

## Abbreviations

ANOVA: Analysis of Variance
BATT: Bilateral Anterior Thigh Thickness
BIA: Bioelectrical Impedance Analysis
BMI: Body Mass Index
CFS: Clinical Frailty Score
CT: Computed Tomography
DXA: Dual X-Ray Absorptiometry
ECW: Extracellular Water
EWGSOP: European Working Group on Sarcopenia in Older People
ICW: Intracellular Water
IQR: Interquartile Range
JVP: Jugular Venous Pressure
MF-BIA: Multi-frequency Bioelectrical Impedance Analysis
MLT: Muscle Layer Thickness
MRI: Magnetic Resonance Imaging
QEHB: Queen Elizabeth Hospital Birmingham
RF: Rectus Femoris
SC: Subcutaneous
SMM: Skeletal Muscle Mass
TBW: Total Body Water
VI: Vastus Intermedius
